# Associations of Myosteatosis with Osteoporosis, Vertebral Fractures, and Long-Term Prognosis in Patients with Cirrhosis

**DOI:** 10.3390/jcm15218438

**Published:** 2026-10-29

**Authors:** Sachie Kiryu, Chisato Saeki, Mitsuru Saito, Tomoya Kanai, Naoto Nishimura, Koichi Oishi, Tomone Sarukawa, Takahiko Toyonaga, Masanori Nakano, Yuichi Torisu, Masayuki Saruta, Akihito Tsubota

**Affiliations:** 1 Division of Gastroenterology and Hepatology, Department of Internal Medicine, The Jikei University School of Medicine, 3-25-8 Nishi-shimbashi, Minato-ku, Tokyo 105-8461, Japan; skiryu63@gmail.com (S.K.); tomoyaaust@hotmail.com (T.K.); naoto3326@gmail.com (N.N.); kooochaan@gmail.com (K.O.); tomone19951122@gmail.com (T.S.); toyo.necco@gmail.com (T.T.); masanori-nakano@jikei.ac.jp (M.N.); torisu@jikei.ac.jp (Y.T.); m.saruta@jikei.ac.jp (M.S.); 2 Division of Gastroenterology, Department of Internal Medicine, Fuji City General Hospital, 50 Takashima-cho, Fuji-shi 417-8567, Shizuoka, Japan; 3 Department of Orthopedic Surgery, The Jikei University School of Medicine, 3-25-8 Nishi-shimbashi, Minato-ku, Tokyo 105-8461, Japan; xlink67@gol.com; 4 Project Research Units, Research Center for Medical Science, The Jikei University School of Medicine, 3-25-8 Nishi-shimbashi, Minato-ku, Tokyo 105-8461, Japan

**Keywords:** cirrhosis, myosteatosis, osteoporosis, vertebral fractures, sarcopenia, prognosis

## Abstract

**Background:** Myosteatosis is increasingly recognized as a clinically relevant skeletal muscle abnormality associated with adverse clinical outcomes. This study aimed to investigate the associations of myosteatosis with osteoporosis, vertebral fractures, and prognosis in patients with cirrhosis. **Methods:** This retrospective study included 139 patients with cirrhosis. Skeletal muscle radiodensity was calculated as the mean Hounsfield unit (HU) at the L3 level on computed tomography. Myosteatosis was defined as <33 HU for men and <28 HU for women, using previously reported sex-specific cutoffs derived from a cohort of patients with cirrhosis. Sarcopenia and osteoporosis were diagnosed according to the Japanese Society of Hepatology and World Health Organization criteria, respectively. Vertebral fractures were assessed using lateral thoracolumbar radiographs. **Results:** The prevalences of myosteatosis, osteoporosis, vertebral fractures, and sarcopenia were 62.6%, 32.4%, 30.9%, and 36.0%, respectively. During a median follow-up of 67.1 months, 37 liver-related deaths (26.6%) occurred. The prevalence of myosteatosis was significantly higher in patients with osteoporosis (*p* = 0.003), vertebral fractures (*p* = 0.021), and sarcopenia (*p* < 0.001) than in those without the respective conditions. The coexistence of myosteatosis and sarcopenia was associated with a higher prevalence of osteoporosis and vertebral fractures than either condition alone or neither condition (*p* < 0.001 for both). Osteoporosis remained independently associated with myosteatosis after multivariable adjustment (adjusted odds ratio = 3.04; *p* = 0.039). Liver-related mortality-free survival was significantly lower in the myosteatosis group than in the non-myosteatosis group (*p* = 0.007). Furthermore, myosteatosis was independently associated with liver-related mortality (adjusted hazard ratio = 2.78; *p* = 0.026). **Conclusions:** Myosteatosis is associated with bone diseases and poorer survival, highlighting the importance of comprehensive musculoskeletal assessment in patients with myosteatosis.

## 1. Introduction

Alterations in body composition, including skeletal muscle, bone, and adipose tissue, are increasingly recognized as major clinical concerns in patients with cirrhosis [1]. These alterations are highly prevalent and are closely associated with adverse clinical outcomes [2,3,4,5]. Beyond sarcopenia, defined by declines in skeletal muscle mass and strength, recent research has highlighted the clinical relevance of muscle quality, particularly myosteatosis [6,7,8]. Myosteatosis is characterized by excessive fat accumulation within skeletal muscle tissue, resulting in impaired muscle metabolism and function [6,9]. Notably, this alteration may represent an early pathological change that precedes other muscle abnormalities, including sarcopenia [10]. A meta-analysis demonstrated a high pooled prevalence of myosteatosis in cirrhosis, estimated at 46% [7]. This condition likely arises from multiple overlapping mechanisms shared with sarcopenia, including aging, physical inactivity (i.e., reduced mechanical loading), chronic inflammation, oxidative stress, hormonal dysregulation, nutritional imbalance (both excess and deficiency), insulin resistance, hyperammonemia, and mitochondrial dysfunction [2,6,8,9]. Clinically, myosteatosis frequently coexists with reduced skeletal muscle mass and impaired physical performance. It is also associated with an increased risk of portal hypertension, hepatic decompensation-related events, and hospitalization. Notably, the concomitant presence of myosteatosis and sarcopenia portends particularly poor clinical outcomes [6,11,12]. In this context, assessment of both muscle quantity and quality may enable earlier identification of patients at increased risk of adverse events.

Osteoporosis and related fractures are also common musculoskeletal complications in patients with cirrhosis [5]. These bone diseases arise from factors inherent to cirrhosis, such as physical inactivity, malnutrition, hormonal imbalance, and elevated inflammatory cytokine levels, and are closely associated with skeletal muscle dysfunction, including sarcopenia and physical frailty [5,13,14,15]. The bidirectional relationship between sarcopenia and bone diseases can create a vicious cycle that progressively worsens the clinical course and prognosis of patients with cirrhosis [15,16]. Previous studies in the general population have demonstrated associations between myosteatosis and both osteoporosis and related fractures [17,18,19,20,21]. However, this relationship remains poorly characterized in patients with cirrhosis, despite growing recognition of the muscle–bone axis.

Therefore, to further elucidate the link between muscle and bone diseases, we investigated the association between myosteatosis and osteoporosis or vertebral fractures (the most common fracture type in this population [15]), and the impact of these conditions on long-term prognosis in patients with cirrhosis.

## 2. Materials and Methods

### 2.1. Study Participants

This retrospective study enrolled 139 consecutive outpatients with cirrhosis managed at Fuji City General Hospital (Shizuoka, Japan) between March 2017 and December 2025. Eligibility criteria included a confirmed diagnosis of cirrhosis; availability of data on bone mineral density (BMD), vertebral fracture status, handgrip strength, and computed tomography (CT)-based skeletal muscle measurements; and no prior treatment for osteoporosis. Patients with malignancies (including hepatocellular carcinoma) at study entry, pathological fractures (e.g., bone metastases or malignancies), or recent prolonged glucocorticoid use (>5 mg/day for >3 months within the preceding year) were excluded. The diagnosis of cirrhosis was established based on integrated laboratory, endoscopic, and imaging findings, as previously described [14]. Hepatic functional reserve was assessed using the Child–Pugh (CP) scoring system [22]. Laboratory parameters, including serum total bilirubin, albumin, creatinine, Mac-2 binding protein glycosylation isomer (M2BPGi; a marker of hepatic fibrosis), and prothrombin time–international normalized ratio (PT–INR) were measured using standard laboratory procedures. The estimated glomerular filtration rate (eGFR) was calculated using the equation developed for Japanese adults by the Japanese Society of Nephrology [23], as follows: eGFR (mL/min/1.73 m^2^) = 194 × serum creatinine^−1.094^ × Age^−0.287^ (×0.739 for women). The primary endpoint was liver-related mortality. Deaths from non-liver-related causes were treated as censoring events, and patients who underwent liver transplantation were censored on the date of transplantation. This study was conducted in accordance with the ethical principles of the 2013 Declaration of Helsinki and approved by the Ethics Committees of the Jikei University School of Medicine (approval number: 37-504) and Fuji City General Hospital (approval number: 406). Consent to participate was obtained through an opt-out procedure. The requirement for written informed consent was waived by the ethics committees due to the retrospective nature of the study.

### 2.2. Sarcopenia and Myosteatosis Assessment

Sarcopenia was diagnosed according to the revised diagnostic criteria proposed by the Japanese Society of Hepatology (second edition) [24,25]. Skeletal muscle area was quantified on CT images at the third lumbar vertebra (L3) level using an attenuation threshold of −29 to +150 Hounsfield units (HU) [26], with dedicated image analysis software (Synapse Vincent, version 7.3; Fujifilm, Tokyo, Japan). The skeletal muscle index (SMI) was calculated by normalizing skeletal muscle area to height squared (cm^2^/m^2^). Sarcopenia was defined as the concomitant presence of low SMI (<42 cm^2^/m^2^ for men and <38 cm^2^/m^2^ for women) and impaired handgrip strength (<28 kg for men and <18 kg for women). Myosteatosis was defined as a mean skeletal muscle radiodensity at the L3 level of <33 HU for men and <28 HU for women (sex-specific criteria derived from a cohort of patients with cirrhosis) [26]. In addition, the most widely used BMI-based criteria established in cancer populations (<33 HU in patients with a BMI ≥ 25 kg/m^2^ and <41 HU in those with a BMI < 25 kg/m^2^) were also applied to evaluate their clinical utility for classifying myosteatosis [27].

### 2.3. Osteoporosis and Fracture Assessment

BMD was measured at the lumbar spine (L2–L4), femoral neck, and total hip using dual-energy X-ray absorptiometry (PRODIGY; GE Healthcare, Chicago, IL, USA). Osteoporosis was defined as a T-score ≤ −2.5 according to the World Health Organization criteria [28]. Vertebral fractures were evaluated on lateral thoracolumbar radiographs using the Genant semiquantitative method and classified according to severity as mild (grade 1), moderate (grade 2), or severe (grade 3) [29].

### 2.4. Statistical Analysis

Categorical variables are presented as frequencies (percentages), and continuous variables as medians (interquartile range). Between-group comparisons were performed using the chi-square test for categorical data and the Mann–Whitney U test for continuous data. The kappa coefficient was used to evaluate the level of agreement between two criteria for classifying myosteatosis. Correlations between skeletal muscle radiodensity and baseline characteristics, including BMD at each measurement site, SMI, and handgrip strength, were evaluated using Spearman’s rank correlation coefficient. To identify factors associated with myosteatosis, univariate logistic regression analyses were performed. Subsequently, variables were selected for inclusion in the multivariable logistic regression model using the forced-entry method, based on clinical relevance and their potential roles as confounders. To minimize model instability and overfitting, the number of covariates was restricted to five, ensuring an events-per-variable (EPV) ratio of ≥10 (myosteatosis, *n* = 87; non-myosteatosis, *n* = 52). The optimal cutoff values of skeletal muscle radiodensity for predicting sarcopenia, osteoporosis, and vertebral fractures were determined using receiver operating characteristic (ROC) curve analysis and the Youden index [30].

Liver-related mortality-free survival was estimated using Kaplan–Meier survival curves and compared via the log-rank test with Bonferroni adjustment for multiple comparisons. These estimates represent cause-specific survival rather than the cumulative incidence of liver-related mortality. To identify factors independently associated with liver-related mortality, univariate Cox proportional hazards regression analyses were performed. Subsequently, variables were selected for inclusion in the multivariable Cox proportional hazards model using the forced-entry method, based on clinical relevance and their potential roles as confounders. Given the relatively small number of liver-related mortality cases (*n* = 37), the number of covariates was restricted to four to reduce the risk of overfitting while maintaining an EPV ratio as close to 10 as possible. Age and sex were included in the multivariable model on the basis of their clinical relevance. To avoid multicollinearity, albumin, total bilirubin, and PT-INR, which are components of the CP score, were not included simultaneously in the multivariable models. Covariate-adjusted survival curves were estimated by applying marginal standardization to the multivariable Cox proportional hazards model.

Statistical analyses were performed using SPSS Statistics version 27.0 (IBM Japan, Tokyo, Japan) and R software (version 4.6.1; R Foundation for Statistical Computing, Vienna, Austria), with a two-sided *p*-value < 0.05 indicating statistical significance.

## 3. Results

### 3.1. Patient Characteristics

The patient selection process is illustrated in Figure S1. Of the 166 patients with cirrhosis initially screened, 27 were excluded according to the exclusion criteria, leaving 139 patients for the final analysis. Table 1. summarizes the baseline characteristics of the 139 participants (76 men [54.7%] and 63 women [45.3%]; median age, 69 [57–76] years). The median CP score was 6 (5–7), and 37.4% of patients (52/139) were classified as CP class B or C. Osteoporosis, vertebral fractures, and sarcopenia were observed in 32.4% (45/139), 30.9% (43/139; grade 1, n = 6; grade 2, n = 16; grade 3, n = 21), and 36.0% (50/139), respectively.

### 3.2. Comparison of Two Criteria for Classifying Myosteatosis

**Figure 1:**
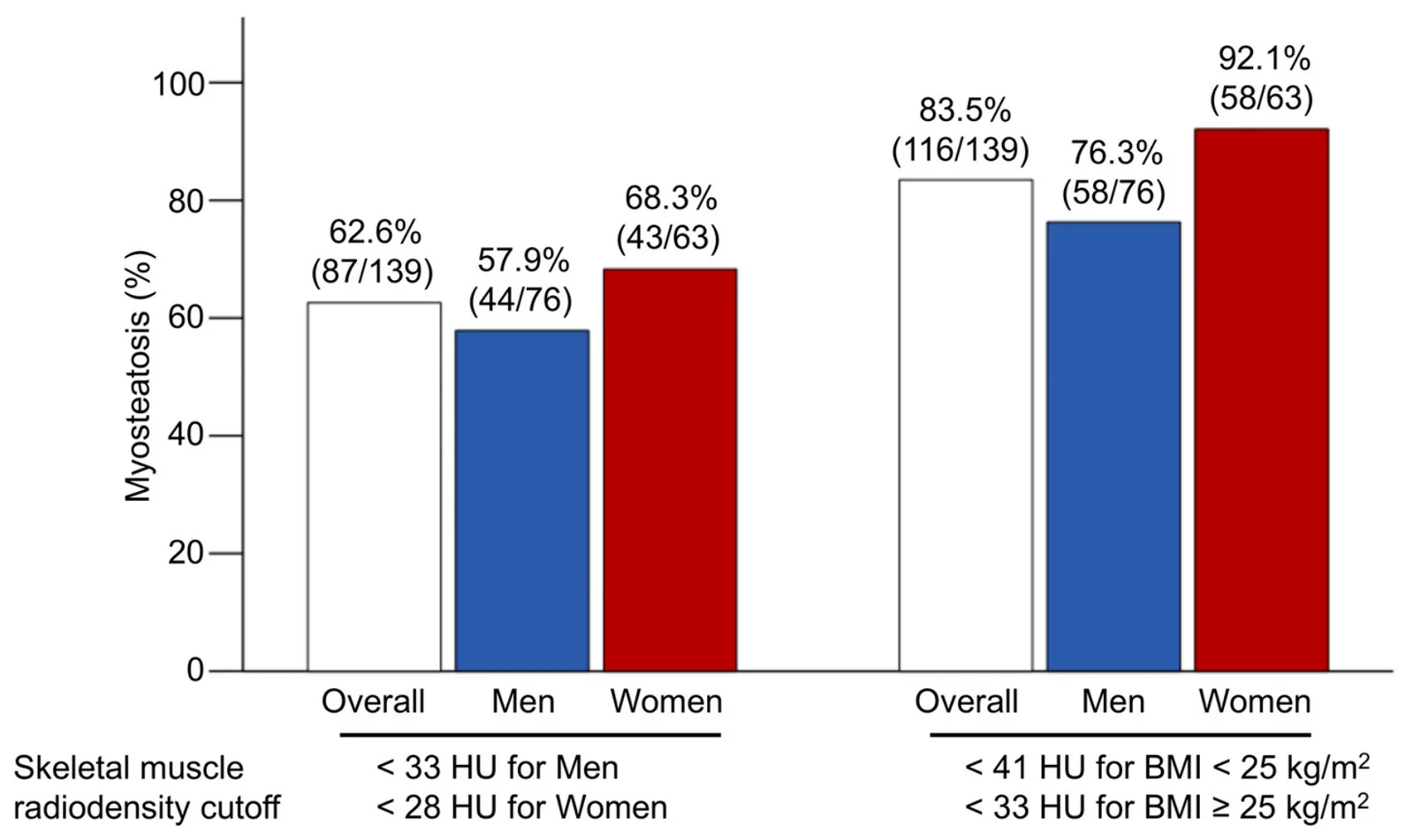
Comparison of the prevalence of myosteatosis between two different criteria. HU, Hounsfield units.

Using the sex-specific criteria, the prevalence of myosteatosis was 62.6% (87/139) overall, 57.9% (44/76) in men, and 68.3% (43/63) in women (Figure 1). In contrast, the BMI-based criteria yielded substantially higher prevalence rates of 83.5% (116/139) overall, 76.3% (58/76) in men, and 92.1% (58/63) in women, with only moderate agreement between the two criteria (kappa coefficient = 0.50). When stratified by BMI (<25 vs. ≥25 kg/m^2^), a marked discrepancy between the two criteria was observed in patients with BMI < 25 kg/m^2^: the prevalence of myosteatosis in this subgroup was 66.7% (58/87) by the sex-specific criteria (Figure S2), compared with 96.6% (84/87) by the BMI-based criteria (Figure S3), indicating that the latter classified virtually all low-BMI patients as having myosteatosis. Given this limitation, the sex-specific criteria were considered more appropriate for the present cohort and were used for all subsequent analyses.

### 3.3. Clinical Characteristics of Patients with and Without Myosteatosis

**Figure 2:**
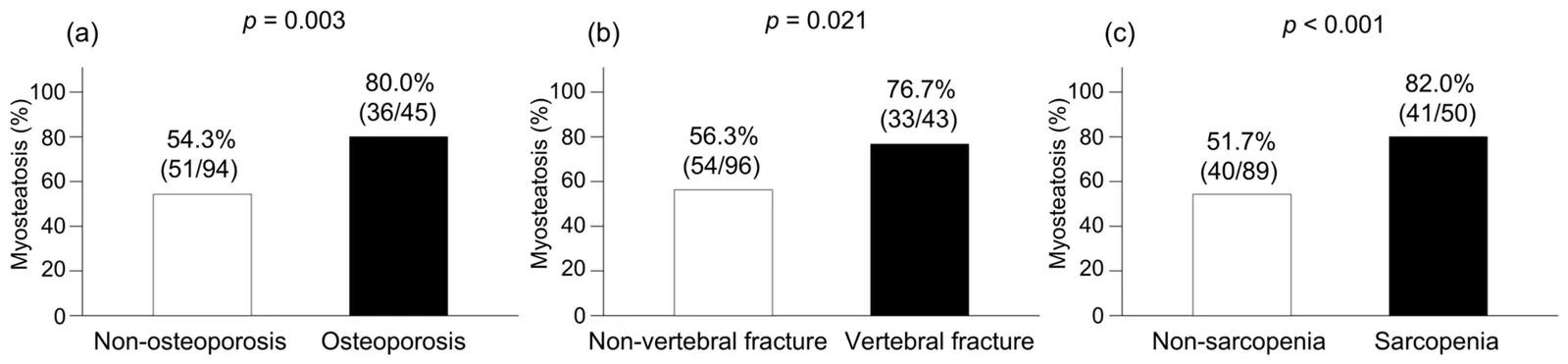
Prevalence of myosteatosis in patients with and without (**a**) osteoporosis, (**b**) vertebral fractures, and (**c**) sarcopenia.

Patients with myosteatosis (myosteatosis group) were significantly older than those without myosteatosis (non-myosteatosis group; *p* < 0.001; Table 1). Compared with the non-myosteatosis group, the myosteatosis group showed higher creatinine levels (*p* = 0.034) and lower eGFR (*p* < 0.001) and albumin levels (*p* = 0.005). The prevalence of myosteatosis was significantly higher in patients with osteoporosis (80.0% vs. 54.3%, *p* = 0.003), vertebral fractures (76.7% vs. 56.3%, *p* = 0.021), and sarcopenia (82.0% vs. 51.7%, *p* < 0.001) than in those without the respective conditions (Figure 2). No significant differences in myosteatosis prevalence were observed among liver disease etiologies (*p* = 0.090; Figure S4).

The 139 participants were then categorized into four groups according to the presence or absence of osteoporosis and sarcopenia: (1) neither condition (54.0% [75/139]), (2) osteoporosis alone (10.1% [14/139]), (3) sarcopenia alone (13.7% [19/139]), and (4) osteosarcopenia (22.3% [31/139]). The prevalence of myosteatosis was significantly higher in the sarcopenia-alone group (84.2% [16/19]; adjusted residual = |2.1|) and the osteosarcopenia group (80.6% [25/31]; adjusted residual = |2.4|) than in the group with neither condition (46.7% [35/75]; adjusted residual = |4.2|; *p* < 0.001; Figure S5).

### 3.4. Association of Skeletal Muscle Radiodensity with BMD, SMI, and Handgrip Strength

**Figure 3:**
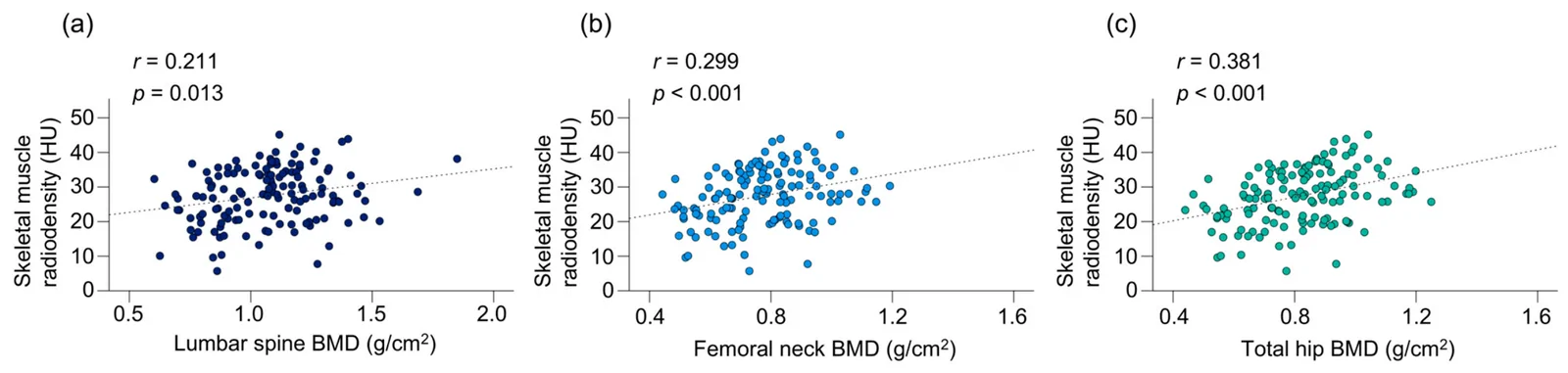
Correlations of skeletal muscle radiodensity with bone mineral density (BMD) at the (**a**) lumbar spine, (**b**) femoral neck, and (**c**) total hip.

**Figure 4:**
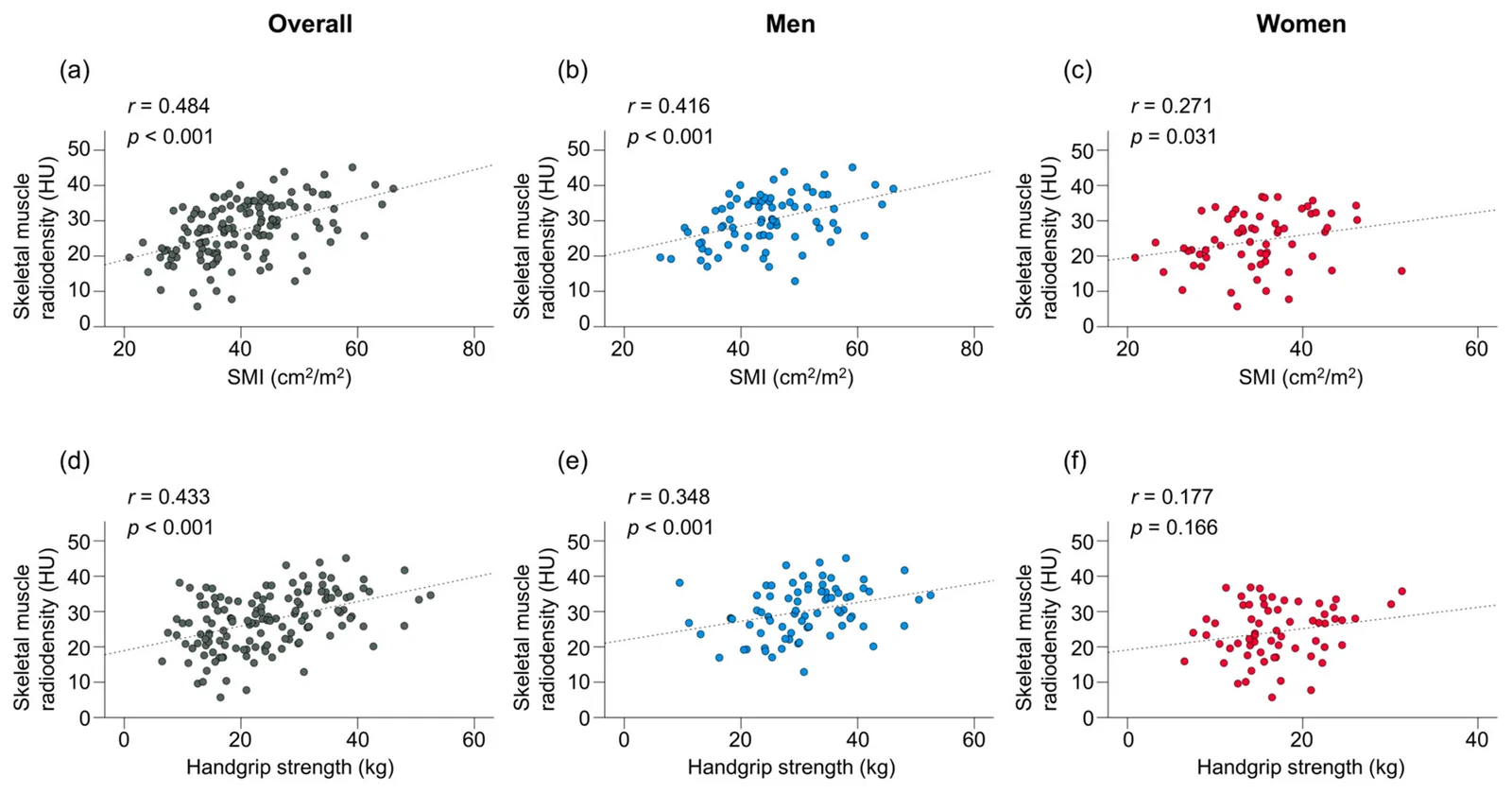
Correlations of skeletal muscle radiodensity with skeletal muscle mass index (SMI) in (**a**) all patients, (**b**) men, and (**c**) women, and with handgrip strength in (**d**) all patients, (**e**) men, and (**f**) women.

Skeletal muscle radiodensity was positively correlated with BMD at the lumbar spine (*r* = 0.211; *p* = 0.013), femoral neck (*r* = 0.299; *p* < 0.001), and total hip (*r* = 0.381; *p* < 0.001; Figure 3). It was also positively correlated with SMI overall (*r* = 0.484; *p* < 0.001), in men (*r* = 0.416; *p* < 0.001), and in women (*r* = 0.271; *p* = 0.031), as well as with handgrip strength overall (*r* = 0.433; *p* < 0.001) and in men (*r* = 0.348; *p* < 0.001), but not in women (*r* = 0.177; *p* = 0.166; Figure 4). Moreover, skeletal muscle radiodensity was significantly correlated with age (*r* = −0.391; *p* < 0.001), albumin (*r* = 0.209; *p* = 0.014), and eGFR (*r* = 0.249; *p* = 0.003; Table S1).

### 3.5. Factors Associated with Myosteatosis

The results of univariate logistic regression analyses are presented in Table S2. Based on clinical relevance, potential confounding effects, and considerations regarding model stability, five variables (age, gender, albumin, eGFR, and osteoporosis) were selected for inclusion in the multivariable logistic regression model. Sarcopenia was excluded because of its substantial overlap with myosteatosis (Figure S5), whereas vertebral fractures were excluded because of their potential association with osteoporosis. Multivariable logistic regression analysis identified lower albumin (adjusted odds ratio [OR] per 1 g/dL increase, 0.243; 95% confidence interval [CI], 0.113–0.525; *p* < 0.001), lower eGFR (adjusted OR per 1 mL/min/1.73 m^2^ increase, 0.971; 95% CI, 0.950–0.993; *p* = 0.011), and presence of osteoporosis (adjusted OR, 3.040; 95% CI, 1.060–8.717; *p* = 0.039) as factors independently associated with myosteatosis (Table 2).

### 3.6. Combined Impacts of Myosteatosis and Sarcopenia on Bone Disease Prevalence

**Figure 5:**
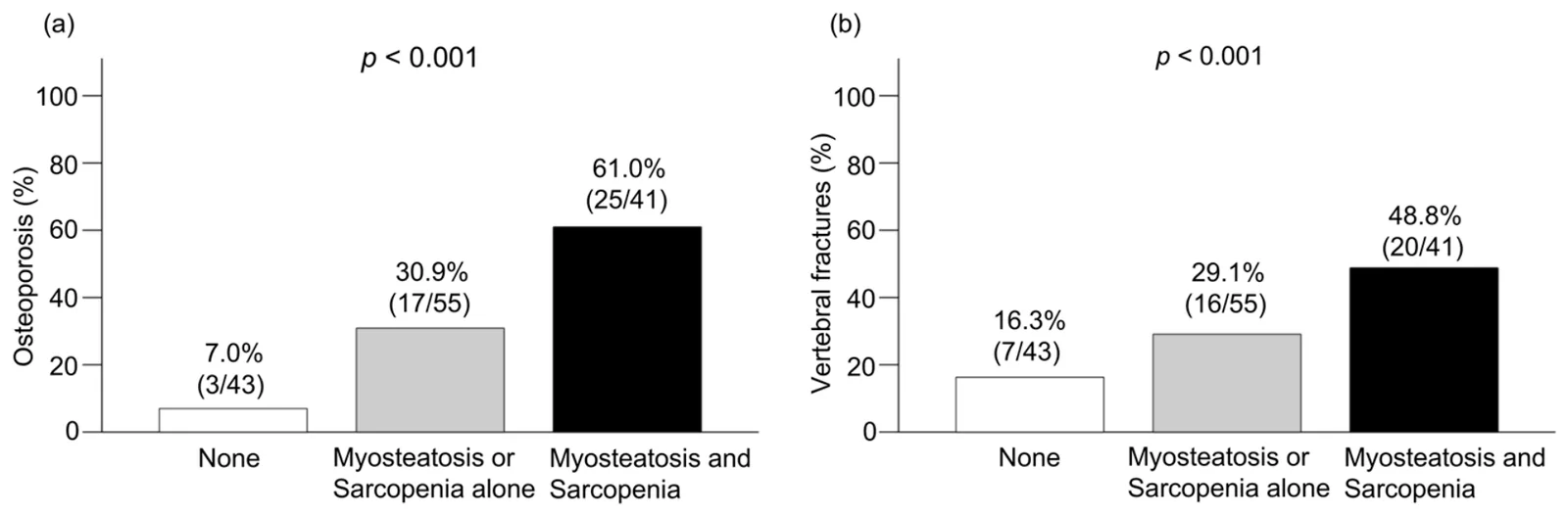
Comparison of the prevalence of (**a**) osteoporosis and (**b**) vertebral fractures among three groups according to the presence of myosteatosis and sarcopenia: (1) neither condition (none), (2) myosteatosis or sarcopenia alone, and (3) both conditions.

To investigate the impact of the coexistence of sarcopenia and myosteatosis on bone disease prevalence, participants were categorized into three groups: (1) neither condition (30.9% [43/139]), (2) either condition alone (39.6% [55/139]), and (3) both conditions (29.5% [41/139]). Osteoporosis (61.0% [25/41]; adjusted residual = |4.7|) and vertebral fractures (48.8% [20/41]; adjusted residual = |2.9|) were most frequent in the group with both conditions and least frequent in the group with neither condition (7.0% [3/43]; adjusted residual = |4.3| for osteoporosis and 16.3% [7/43]; adjusted residual = |2.5| for vertebral fractures; Figure 5) (*p* < 0.001 for the prevalences of osteoporosis and vertebral fractures).

### 3.7. Optimal Cutoff Values of Skeletal Muscle Radiodensity for Predicting Sarcopenia, Osteoporosis, and Vertebral Fractures

**Figure 6:**
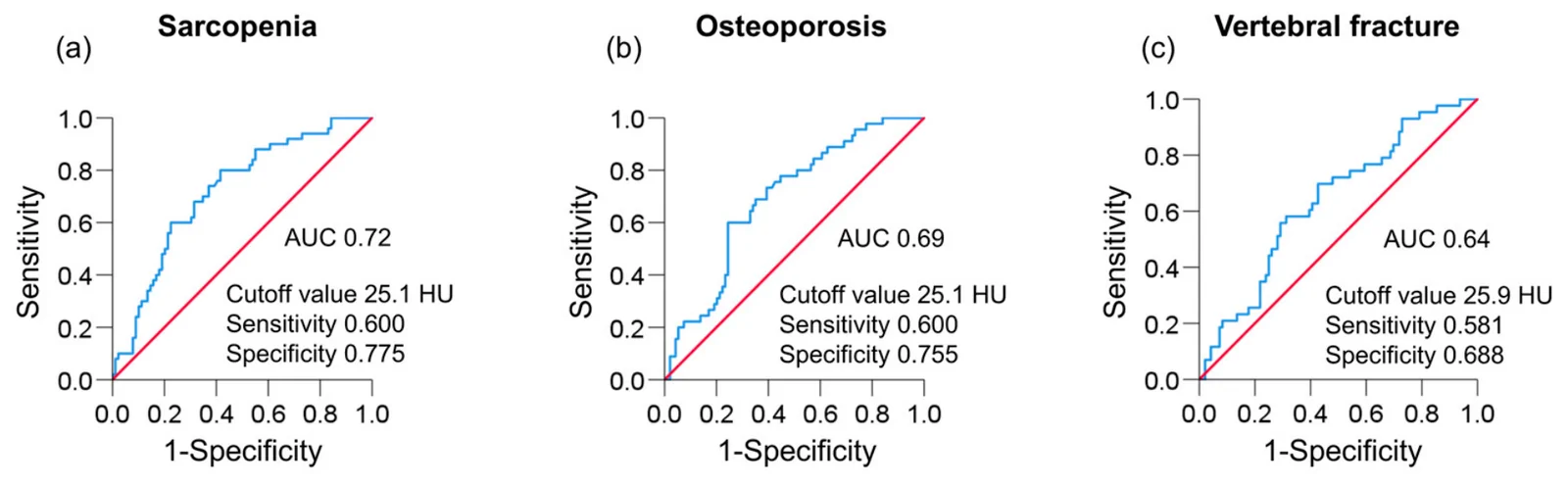
The receiver operating characteristic analysis of skeletal muscle radiodensity for predicting sarcopenia, osteoporosis, and vertebral fractures. (**a**) The cutoff value for sarcopenia was 25.1 Hounsfield units (HU) with an area under the curve (AUC), sensitivity, and specificity of 0.72, 0.600, and 0.775, respectively. (**b**) The cutoff value for osteoporosis was 25.1 HU, with AUC, sensitivity, and specificity of 0.69, 0.600, and 0.755, respectively. (**c**) The cutoff value for vertebral fractures was 25.9 HU, with AUC, sensitivity, and specificity of 0.64, 0.581, and 0.688, respectively.

Given the close associations of myosteatosis with sarcopenia and bone diseases, we evaluated the optimal cutoff values of skeletal muscle radiodensity for predicting sarcopenia, osteoporosis, and vertebral fractures using ROC curve analysis (Figure 6). The cutoff values were 25.1HU [area under the curve (AUC), 0.72; sensitivity/specificity, 0.600/0.775] for sarcopenia, 25.1 HU (0.69; 0.600/0.755) for osteoporosis, and 25.9 HU (0.64; 0.581/0.688) for vertebral fractures.

### 3.8. Comparison of Cumulative Liver-Related Mortality-Free Survival Between the Myosteatosis and Non-Myosteatosis Groups

**Figure 7:**
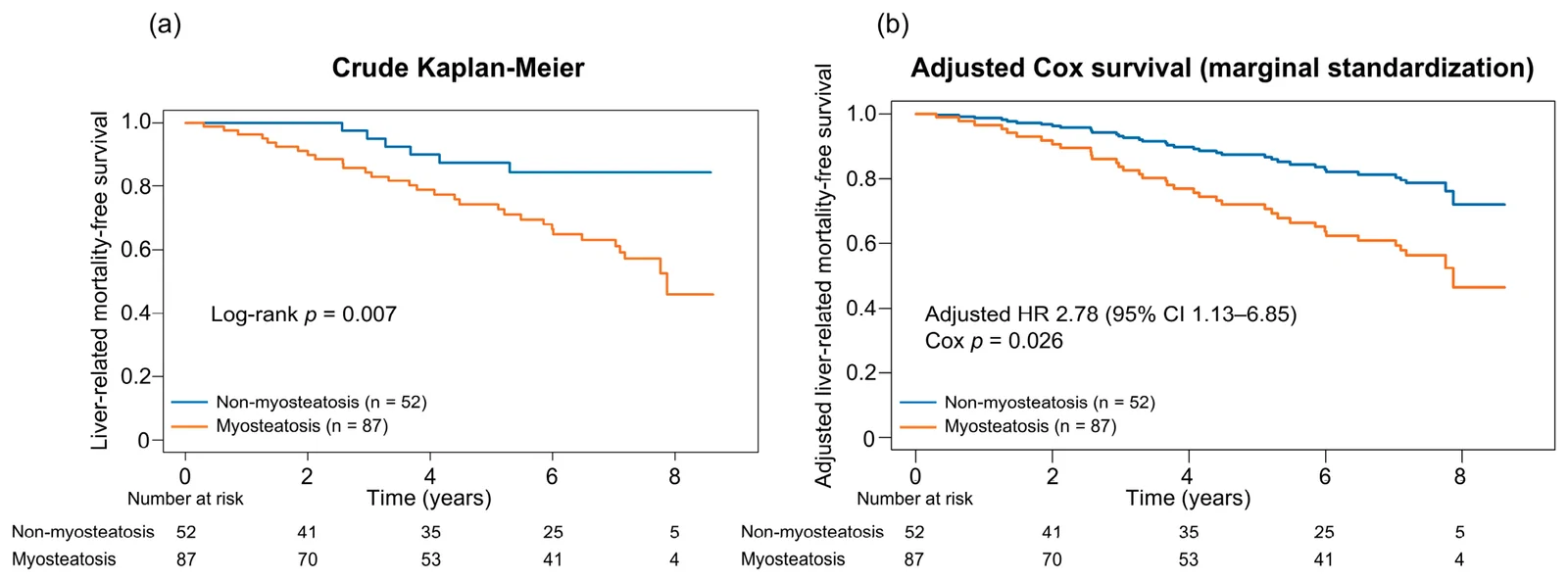
Unadjusted Kaplan–Meier curves (**a**) and covariate-adjusted Cox survival curves (**b**) for liver-related mortality-free survival according to myosteatosis status.

During the observation period (median, 67.1 [30.7–88.3] months), 37 patients (26.6%) died of liver-related causes (liver failure, *n* = 25; incident HCC, *n* = 7; rupture of esophageal varices, *n* = 4; and spontaneous bacterial peritonitis, *n* = 1). Kaplan–Meier analysis demonstrated significantly lower liver-related mortality-free survival in patients with myosteatosis than in those without myosteatosis (log-rank *p* = 0.007; Figure 7a).

Next, the 139 patients were stratified into CP class A and class B/C, and cumulative survival rates were compared between the myosteatosis and non-myosteatosis groups (Figure S6). In both the CP class A and class B/C subgroups, liver-related mortality-free survival rates were numerically lower in patients with myosteatosis than in those without myosteatosis; however, neither difference reached statistical significance (class A, *p* = 0.076; class B/C, *p* = 0.051).

To further investigate the combined impact of musculoskeletal abnormalities, survival analyses were performed by stratifying patients into four groups according to the presence or absence of myosteatosis in combination with osteoporosis (Figure S7), vertebral fractures (Figure S8), or sarcopenia (Figure S9). Across all analyses, patients with both myosteatosis and osteoporosis (*p* = 0.022), myosteatosis and vertebral fractures (*p* = 0.006), or myosteatosis and sarcopenia (*p* < 0.001) had significantly worse survival than those with neither condition. In each analysis, patients with only one of the two conditions showed intermediate survival between those with neither condition and those with both conditions; however, these differences did not reach statistical significance.

### 3.9. Prognostic Factors in Patients with Cirrhosis

The results of univariate Cox proportional hazards analyses are presented in Table S3. Four variables (age, gender, CP score, and myosteatosis) were selected for inclusion in the multivariable Cox proportional hazards model on the basis of clinical relevance and potential confounding effects, while considering the limited number of liver-related mortality. Multivariable Cox proportional hazards regression analysis identified older age (adjusted hazard ratio [HR] per 1-year increase, 1.039; 95% CI, 1.003–1.077; *p* = 0.036), higher CP score (adjusted HR per 1-point increase, 1.679; 95% CI, 1.317–2.140; *p* < 0.001), and the presence of myosteatosis (adjusted HR, 2.782; 95%CI, 1.129–6.854; *p* = 0.026) as factors independently associated with liver-related mortality (Table 3). Covariate-adjusted survival curves derived using marginal standardization of the multivariable Cox model showed lower estimated liver-related mortality-free survival in patients with myosteatosis than in those without myosteatosis (Figure 7b).

## 4. Discussion

In patients with cirrhosis, muscle and bone disorders are closely interrelated and exert synergistic effects on adverse clinical outcomes [14,15,16]. Recent studies have highlighted myosteatosis as a clinically relevant feature of impaired skeletal muscle quality that may be associated with musculoskeletal dysfunction [6,7,8]. As outlined in the Introduction, myosteatosis and bone diseases likely share several pathophysiological mechanisms, suggesting a close biological relationship between the two conditions. While population-based studies have demonstrated associations between myosteatosis and osteoporosis or fractures [17,18,19,20,21], this relationship has not been fully elucidated in patients with cirrhosis. To our knowledge, this is the first study to examine the relationship between myosteatosis and bone diseases, as well as their associations with long-term prognosis, in a cirrhotic population. Our findings demonstrated that skeletal muscle radiodensity was positively correlated with BMD, SMI, and handgrip strength. In addition, myosteatosis was significantly more prevalent in patients with osteoporosis, vertebral fractures, or sarcopenia than in those without the respective conditions. The optimal cutoff values of skeletal muscle radiodensity for identifying these conditions were approximately 25–26 HU; however, the modest discriminatory ability (AUC, 0.64–0.72) suggests that the clinical utility of these cutoffs is limited and requires further validation. Multivariable analysis identified osteoporosis as an independent factor associated with myosteatosis.

The coexistence of myosteatosis (reflecting poor muscle quality) and sarcopenia (reflecting reduced muscle quantity) was associated with a higher prevalence of bone diseases than either condition alone or neither. Furthermore, myosteatosis was independently associated with mortality after adjustment for potential confounders (age, sex, and CP score).

Previous studies provide mechanistic insights supporting our findings. One study reported that patients with osteoporosis at the lumbar spine or femoral neck exhibited significantly greater fatty infiltration of the psoas muscle, particularly at the upper lumbar levels, indicating a potential link between myosteatosis and osteoporosis [17]. Another study identified the bone load index and fatty infiltration of the paraspinal and right psoas major muscles as independent factors associated with vertebral fractures [18]. Interaction analyses further demonstrated a significant interaction between bone load index and muscle fat infiltration, suggesting that progressive muscle degeneration may amplify the impact of vertebral load-strength imbalance on fracture risk. Consistently, thigh muscle fatty infiltration was significantly associated with hip fracture risk, independent of BMD [20]. Collectively, these findings support the concept that muscle quality, alongside muscle mass, may be associated with skeletal integrity through mechanical loading and shared pathophysiological processes within the muscle–bone axis [20].

In this study, skeletal muscle radiodensity was correlated with age, albumin, and eGFR, all of which were significantly associated with myosteatosis. Albumin and eGFR remained independently associated with myosteatosis in multivariable analysis. Aging facilitates alterations in fat distribution and intramyocellular fat accumulation, potentially mediated by age-related changes in muscle stem cell differentiation toward adipocytes [31]. Hypoalbuminemia reflects poor nutritional status and systemic inflammation, both of which can promote muscle catabolism and ectopic fat deposition [9,32]. Impaired kidney function is also associated with increased skeletal muscle fat accumulation, paralleling renal function decline and potentially reversible following functional restoration [33]. The underlying mechanisms are likely multifactorial, including chronic inflammation, insulin resistance, uremic toxin accumulation, and mitochondrial dysfunction [33,34]. Given the bidirectional interaction within the liver–kidney axis [35], renal impairment may be an important factor associated with myosteatosis in patients with cirrhosis. Taken together, these findings suggest that myosteatosis may reflect systemic metabolic and inflammatory disturbances inherent to cirrhosis, rather than a localized change in skeletal muscle.

Standardized cutoff values for myosteatosis in liver disease remain to be established. Consequently, this study compared two previously reported criteria: BMI-based thresholds originally established in Western cancer populations and sex-specific criteria derived from patients with cirrhosis [26,27]. Application of BMI-based criteria resulted in a prevalence exceeding 80% in the overall cohort (consistent with previous reports in Japanese populations [36]), with virtually all patients with a BMI < 25 kg/m^2^ classified as having myosteatosis. BMI values in the present cohort appeared lower than those reported in the Western cancer cohorts [27]. Furthermore, at equivalent BMI values, individuals of Asian ancestry tend to have higher body fat percentages and greater intramyocellular lipid content than Caucasians [37], suggesting the BMI-based cutoff of 41 HU for patients with BMI < 25 kg/m^2^ may be disproportionately stringent for Japanese patients. Moreover, BMI-based criteria may be inherently limited in cirrhosis, as body weight can be confounded by fluid retention, such as ascites and edema, and these thresholds do not account for sex-related dimorphism in skeletal muscle lipid storage capacity. For these reasons, sex-specific criteria were adopted for the present study [9]. However, these cutoff values have not been adequately validated across diverse cirrhotic populations, and their applicability and generalizability remain uncertain. Future large-scale, multicenter studies are needed to validate these cutoff values and establish optimal thresholds for myosteatosis, considering potential differences in ethnicity and liver disease etiology.

Musculoskeletal disorders, including sarcopenia, osteosarcopenia, and fractures, are well-recognized prognostic factors in cirrhosis, independent of hepatic reserve function [15,16]. Meanwhile, a recent meta-analysis demonstrated significantly lower survival rates in patients with myosteatosis than in those without (pooled mortality rate: 40% vs. 12%) [7]. In stratified survival analyses, significantly lower liver-related mortality-free survival was observed only when myosteatosis coexisted with osteoporosis, vertebral fractures, or sarcopenia, compared with those with neither condition. Taken together, these findings suggest that myosteatosis may be a clinically relevant prognostic factor among musculoskeletal disorders in patients with cirrhosis. Given that CT imaging is routinely performed in clinical management of cirrhosis, assessment of myosteatosis may provide additional value for risk stratification, not only for bone diseases but also for prognosis.

This study had several limitations. First, vertebral fractures were evaluated based on thoracolumbar radiographs and clinical interviews; consequently, the exact timing of fracture onset could not be determined in some cases. Second, detailed data on nutritional status, vitamin D levels, physical activity, hormonal profiles, and inflammatory markers were unavailable; therefore, residual confounding by these factors cannot be excluded. Third, standardized methodology and diagnostic criteria for myosteatosis have not yet been established. Fourth, the present cohort was relatively small and geographically restricted, which may have introduced unidentified bias. In addition, although the multivariable Cox proportional hazards model included only four covariates, the relatively small number of liver-related deaths may have increased the risk of overfitting. Finally, the retrospective study design and cross-sectional assessment precluded determination of the temporal relationship between myosteatosis and bone diseases; thus, causal relationships cannot be established from the present findings. Therefore, larger prospective multicenter studies are warranted to validate our findingsand elucidate the longitudinal associations among skeletal muscle fat infiltration, bone-related outcomes, and prognosis.

## 5. Conclusions

In patients with cirrhosis, myosteatosis was more prevalent among those with osteoporosis, vertebral fractures, or sarcopenia, and osteoporosis remained independently associated with myosteatosis in multivariable analysis. Myosteatosis was also independently associated with liver-related mortality. These findings suggest that comprehensive evaluation of musculoskeletal complications, including bone diseases, may be warranted in patients with cirrhosis and myosteatosis.

## Figures and Tables

**Table 1:** Comparison of clinical characteristics between patients with and without myosteatosis.

Variable	All Patients	Myosteatosis	Non-Myosteatosis	*p* Value
Patients, n (%)	139	87 (62.6)	52 (37.4)	
Men/Women, n (%)	76 (54.7)/63 (45.3)	44 (50.6)/43 (49.4)	32 (61.5)/20 (38.5)	0.209
Age (years)	69.0 (57.0–76.0)	73.0 (61.0–78.0)	64.0 (53.3–71.8)	<0.001
BMI (kg/m^2^)	23.7 (21.2–26.0)	23.2 (20.3–26.0)	24.2 (22.2–26.2)	0.201
Diabetes mellitus, n (%)	43 (30.9)	25 (28.7)	18 (34.6)	0.468
HBV/HCV/Alcohol/PBC/MASH/other, n	11/36/49/12/17/14	6/27/25/6/11/12	5/9/24/6/6/2	0.090
Child-Pugh score	6 (5–7)	6 (5–7)	6 (5–7)	0.315
Child-Pugh B/C, n (%)	52 (37.4)	36 (41.4)	16 830.8)	0.211
Total bilirubin (mg/dL)	0.9 (0.6–1.3)	0.8 (0.6–1.3)	1.0 (0.6–1.4)	0.591
Albumin (g/dL)	3.8 (3.4–4.2)	3.7 (3.2–4.1)	4.0 (3.5–4.4)	0.005
Prothrombin time INR	1.11 (1.04–1.27)	1.11 (1.06–1.23)	1.13 (1.02–1.35)	0.976
Creatinine (mg/dL)	0.8 (0.7–1.1)	0.8 (0.7–1.1)	0.8 (0.6–0.9)	0.034
eGFR (mL/min/1.73m^2^)	64.0 (50.0–78.0)	56.0 (45.0–74.0)	72.5 (57.3–84.5)	<0.001
CKD	61 (43.9)	46 (52.9)	15 (28.8)	0.006
Sodium (mEq/L)	140 (138–141)	140 (138–141)	140 (138–141)	0.306
Platelet (×10^4^/µL)	11.8 (8.6–16.5)	11.6 (8.5–15.6)	12.0 (8.9–17.2)	0.385
M2BPGi (C.O.I.)	3.28 (1.48–6.28)	3.32 (1.74–6.25)	2.40 (1.15–6.35)	0.321
Lumbar spine BMD (g/cm^2^)	1.08 (0.89–1.20)	1.06 (0.87–1.23)	1.10 (0.99–1.20)	0.181
Femoral neck BMD (g/cm^2^)	0.77 (0.66–0.87)	0.72 (0.62–0.86)	0.79 (0.72–0.89)	0.025
Total hip BMD (g/cm^2^)	0.83 (0.70–0.93)	0.77 (0.65–0.91)	0.88 (0.77–0.97)	0.001
Osteoporosis, n (%)	45 (32.4)	36 (41.4)	9 (17.3)	0.003
Vertebral fractures, n (%)	43 (30.9)	33 (37.9)	10 (19.2)	0.021
grade: 1/2/3	6/16/21	3/11/19	3/5/2	0.074
SMI (cm^2^/m^2^)				
All patients	38.6 (33.4–45.1)	35.8 (32.8–43.5)	43.2 (38.0–48.6)	<0.001
Men	44.0 (38.1–49.3)	43.3 (34.7–45.9)	46.3 (42.5–53.6)	0.002
Women	34.6 (30.6–38.4)	34.0 (28.9–35.9)	37.0 (32.6–41.4)	0.012
Handgrip strength (kg)				
All patients	23.5 (16.3–31.5)	21.5 (15.0–29.2)	29.3 (18.3–35.3)	0.001
Men	30.7 (25.0–36.6)	28.9 (23.4–32.7)	34.3 (29.1–38.1)	0.003
Women	16.1 (13.9–21.0)	15.6 (13.5–21.0)	16.8 (14.3–23.3)	0.118
Sarcopenia, n (%)	50 (36.0)	41 (47.1)	9 (17.3)	<0.001
Skeletal muscle radiodensity (HU)	27.9 (21.4–33.9)	23.4 (19.4–27.4)	34.9 (33.4–37.4)	<0.001

Values are shown as median (interquartile range) or number (percentage). Statistical analysis was performed using the chi-squared test or the Mann–Whitney U test, as appropriate. BMD, bone mineral density; BMI, body mass index; CKD, chronic kidney disease; C.O.I., cutoff index; eGFR, estimated glomerular filtration rate; HBV, hepatitis B virus; HCV, hepatitis C virus; HU, Hounsfield unit; INR, international normalized ratio; M2BPGi, Mac-2 binding protein glycosylation isomer; MASH, metabolic dysfunction-associated steatohepatitis; PBC, primary biliary cholangitis; SMI, skeletal muscle mass index.

**Table 2:** Factors associated with myosteatosis in patients with cirrhosis.

Variable	Crude		Adjusted	
	OR (95% CI)	*p* Value	OR (95% CI)	*p* Value
Age (per 1-year increase)	1.056 (1.023–1.090)	<0.001	1.037 (0.994–1.082)	0.091
Male sex (vs. female)	0.640 (0.318–1.287)	0.210	0.988 (0.418–2.339)	0.979
Osteoporosis (yes vs. no)	3.373 (1.462–7.778)	0.004	3.040 (1.060–8.717)	0.039
Albumin (per 1 g/dL increase)	0.396 (0.211–0.744)	0.004	0.243 (0.113–0.525)	<0.001
eGFR (per 1 mL/min/1.73 m^2^ increase)	0.969 (0.952–0.987)	<0.001	0.971 (0.950–0.993)	0.011

CI, confidence interval; eGFR, estimated glomerular filtration rate; OR, odds ratio.

**Table 3:** Factors associated with mortality in patients with cirrhosis.

Variable	Crude		Adjusted	
	HR (95% CI)	*p* Value	HR (95% CI)	*p* Value
Age (per 1-year increase)	1.021 (0.990–1.052)	0.181	1.039 (1.003–1.077)	0.036
Male sex (vs. female)	0.739 (0.387–1.408)	0.358	0.939 (0.485–1.819)	0.852
Myosteatosis (yes vs. no)	3.144 (1.311–7.537)	0.010	2.782 (1.129–6.854)	0.026
Child-Pugh score (per 1-point increase)	1.438 (1.175–1.761)	<0.001	1.679 (1.317–2.140)	<0.001

CI, confidence interval; HR, hazard ratio.

## Data Availability

The data generated and analyzed during the current study are not publicly available due to ethical restrictions imposed by the Ethics Committees of the Jikei University School of Medicine and Fuji City General Hospital. The data are available from the corresponding author upon reasonable request.

## Supplementary Materials

The following supporting information can be downloaded at: https://www.mdpi.com/article/10.3390/jcm15218438/s1, Supplementary Table S1: Correlation between skeletal muscle attenuation and baseline characteristics; Supplementary Table S2: Univariate analysis for factors associated with myosteatosis; Supplementary Table S3: Univariate analysis for factors associated with liver-related mortality; Figure S1. Flow diagram of the patient screening process; Figure S2. Prevalence of myosteatosis based on sex-specific criteria stratified by body mass index (BMI < 25 vs. ≥ 25 kg/m^2^); Figure S3. Prevalence of myosteatosis based on body mass index (BMI)-based criteria (BMI < 25 vs. ≥ 25 kg/m^2^); Figure S4. Prevalence of myosteatosis according to liver disease etiology. HBV, hepatitis B virus; HCV, hepatitis C virus; MASH, metabolic dysfunction-associated steatohepatitis; PBC, primary biliary cholangitis; Figure S5. Comparison of the prevalence of myosteatosis among four groups according to the presence of osteoporosis and sarcopenia: (1) neither condition, (2) osteoporosis alone, (3) sarcopenia alone, and (4) both conditions; Figure S6. Comparison of cumulative survival rates between patients with and without myosteatosis stratified by Child–Pugh class: (a) Child–Pugh A and (b) Child–Pugh B/C; Figure S7. Comparison of cumulative survival rates among four groups according to the presence of myosteatosis and osteoporosis: (1) neither condition (none), (2) myosteatosis alone, (3) osteoporosis alone, and (4) both conditions; Figure S8. Comparison of cumulative survival rates among four groups according to the presence of myosteatosis and vertebral fractures: (1) neither condition (none), (2) myosteatosis alone, (3) vertebral fractures alone, and (4) both conditions; Figure S9. Comparison of cumulative survival rates among four groups according to the presence of myosteatosis and sarcopenia: (1) neither condition (none), (2) myosteatosis alone, (3) sarcopenia alone, and (4) both conditions.

## Abbreviations

BMD, bone mineral density; BMI, body mass index; CI, confidence interval; CP, Child–Pugh; CT, computed tomography; eGFR, estimated glomerular filtration rate; EPV, events-per-variable; HR, hazard ratio; HU, Hounsfield unit; INR, international normalized ratio; M2BPGi, Mac-2 binding protein glycosylation isomer; OR, odds ratio; PT–INR, prothrombin time–international normalized ratio; SMI, skeletal muscle mass index.
