# Supplementary material for: Associations of Myosteatosis with Osteoporosis, Vertebral Fractures, and Long-Term Prognosis in Patients with Cirrhosis
Source: J Clin Med. 2026 Oct 29;15(21):8438. doi: 10.3390/jcm15218438 (PMC13764208; doi:10.3390/jcm15218438)
Supplement: Supplementary file 1 [file jcm-15-08438-s001.zip › Supplementary Figure Legends.pdf]

Supplementary Figure Legends

Figure S1. Flow diagram of the patient screening process.

Figure S2. Prevalence of myosteatosis based on sex-specific criteria stratified by body mass index (BMI < 25 vs. ≥ 25 kg/m²).

Figure S3. Prevalence of myosteatosis based on body mass index (BMI)-based criteria (BMI < 25 vs. ≥ 25 kg/m²).

Figure S4. Prevalence of myosteatosis according to liver disease etiology. HBV, hepatitis B virus; HCV, hepatitis C virus; MASH, metabolic dysfunction-associated steatohepatitis; PBC, primary biliary cholangitis.

Figure S5. Comparison of the prevalence of myosteatosis among four groups according to the presence of osteoporosis and sarcopenia: (1) neither condition, (2) osteoporosis alone, (3) sarcopenia alone, and (4) both conditions.

Figure S6. Comparison of cumulative survival rates between patients with and without myosteatosis stratified by Child–Pugh class: (**a**) Child–Pugh A and (**b**) Child–Pugh B/C.

Figure S7. Comparison of cumulative survival rates among four groups according to the presence of myosteatosis and osteoporosis: (1) neither condition (none), (2) myosteatosis alone, (3) osteoporosis alone, and (4) both conditions.

Figure S8. Comparison of cumulative survival rates among four groups according to the presence of myosteatosis and vertebral fractures: (1) neither condition (none), (2) myosteatosis alone, (3) vertebral fractures alone, and (4) both conditions.

Figure S9. Comparison of cumulative survival rates among four groups according to the presence of myosteatosis and sarcopenia: (1) neither condition (none), (2) myosteatosis alone, (3) sarcopenia alone, and (4) both conditions.
