# Supplementary material for: Associations of Myosteatosis with Osteoporosis, Vertebral Fractures, and Long-Term Prognosis in Patients with Cirrhosis
Source: J Clin Med. 2026 Oct 29;15(21):8438. doi: 10.3390/jcm15218438 (PMC13764208; doi:10.3390/jcm15218438)
Supplement: Supplementary file 1 [file jcm-15-08438-s001.zip › Supplementary Tables.pdf]

Table S1. Correlation between skeletal muscle attenuation and baseline characteristics.

Variable	Correlation coefficient	p value
Age (years)	−0.391	< 0.001
BMI (kg/m^2)	0.096	0.261
Child-Pugh score	−0.028	0.747
Total bilirubin (mg/dL)	0.129	0.131
Albumin (g/dL)	0.209	0.014
Prothrombin time INR	0.090	0.295
Creatinine (mg/dL)	−0.050	0.562
eGFR (mL/min/1.73m^2)	0.249	0.003
Sodium (mEq/L)	−0.123	0.147
Platelet (x10^4/μL)	0.008	0.922
M2BPGi (C.O.I.)	−0.056	0.514

BMI, body mass index; C.O.I., cutoff index; eGFR, estimated glomerular filtration rate; INR, international normalized ratio; M2BPGi, Mac-2 binding protein glycosylation isomer.

Table S2. Univariate analysis for factors associated with myosteatosis.

Variable	OR (95% CI)	p value
Gender (Men)	0.640 (0.318–1.287)	0.210
Age (years)	1.056 (1.023–1.090)	< 0.001
BMI (kg/m^2)	0.954 (0.881–1.032)	0.242
Diabetes mellitus	0.762 (0.365–1.590)	0.469
Etiology	1.050 (0.828–1.333)	0.687
Child-Pugh score	1.111 (0.898–1.375)	0.332
Total bilirubin (mg/dL)	1.044 (0.734–1.486)	0.810
Albumin (g/dL)	0.396 (0.211–0.744)	0.004
Prothrombin time INR	0.548 (0.077–3.899)	0.548
Creatinine (mg/dL)	4.654 (1.247–17.305)	0.022
eGFR (mL/min/1.73m^2)	0.969 (0.952–0.987)	<0.001
CKD	2.767 (1.330–5.759)	0.006
Sodium (mEq/L)	1.070 (0.932–1.228)	0.335
Platelet (×10^4/μL)	0.962 (0.908–1.018)	0.181
M2BPGi (C.O.I)	0.991 (0.903–1.088)	0.850
Osteoporosis	3.373 (1.462–7.778)	0.004
Vertebral fracture	2.567 (1.137–5.795)	0.023
Sarcopenia	4.258 (1.852–9.792)	< 0.001

BMI, body mass index; CI, confidence interval; CKD, chronic kidney disease, C.O.I., cutoff index; eGFR, estimated glomerular filtration rate; INR, international normalized ratio; M2BPGi, Mac-2 binding protein glycosylation isomer; OR, odds ratio.

Table S3. Univariate analysis for factors associated with liver-related mortality.

Variable	HR (95% CI)	*p* value
Gender (Men)	0.739 (0.387–1.408)	0.358
Age (years)	1.021 (0.990–1.052)	0.181
BMI (kg/m^2)	0.902 (0.825–0.986)	0.023
Diabetes mellitus	1.321 (0.660–2.646)	0.432
Etiology	1.017 (0.822–1.259)	0.877
Child-Pugh score	1.438 (1.175–1.761)	< 0.001
Total bilirubin (mg/dL)	1.886 (1.357–2.621)	< 0.001
Albumin (g/dL)	0.291 (0.162–0.523)	< 0.001
Prothrombin time INR	4.287 (0.542–33.881)	0.168
Creatinine (mg/dL)	1.926 (0.850–4.365)	0.116
eGFR (mL/min/1.73m^2)	0.992 (0.976–1.008)	0.309
CKD	0.899 (0.470–1.718)	0.747
Sodium (mEq/L)	0.971 (0.835–1.129)	0.703
Platelet (×10^4/μL)	0.926 (0.868–0.989)	0.021
M2BPGi (C.O.I)	1.111 (1.030–1.199)	0.006
Osteoporosis	1.677 (0.878–3.203)	0.117
Vertebral fracture	1.903 (0.985–3.676)	0.055
Sarcopenia	3.014 (1.569–5.788)	< 0.001
Myosteatosis	3.144 (1.311–7.537)	0.010

BMI, body mass index; CI, confidence interval; CKD, chronic liver disease, C.O.I., cutoff index; eGFR, estimated glomerular filtration rate; HR, hazard ratio; INR, international normalized ratio; M2BPGi, Mac-2 binding protein glycosylation isomer.
